# Minocycline-Induced Hyperpigmentation of Nails

**DOI:** 10.7759/cureus.38640

**Published:** 2023-05-06

**Authors:** Kevin Varghese, Jordan Dykstra, Elizabeth Bisbee

**Affiliations:** 1 School of Medicine, University of Missouri at Kansas City, Kansas City, USA; 2 Department of Dermatology, University of Kansas Medical Center, Kansas City, USA

**Keywords:** acne rosacea, nails, type ii hyperpigmentation, drug-induced hyperpigmentation, minocycline hyperpigmentation

## Abstract

Minocycline is an antibiotic used for several dermatologic conditions, including rosacea. The development of skin, scleral, and nail hyperpigmentation may occur with long-term use of minocycline, and this is associated with no adverse effect on function. We present a case of a 66-year-old male who developed blue-gray hyperpigmentation of his nail beds after treating rosacea with systemic minocycline for over 20 years. The remainder of the physical exam was unremarkable for hyperpigmentation elsewhere. The patient was informed that this was likely an adverse effect of his chronic minocycline use. He insisted upon the continuation of minocycline, so he was counseled on the adverse effects of the medication and scheduled for follow-up.

## Introduction

Minocycline is a semisynthetic tetracycline antibiotic used for both its antibacterial and anti-inflammatory activity. These characteristics make minocycline a therapeutic option for several dermatologic conditions, including acne and rosacea [1]. Long-term use of minocycline is associated with skin, scleral, and nail hyperpigmentation, with no adverse effect on function [2-5]. We present a case of a 66-year-old male who developed blue-gray hyperpigmentation of his nail beds after the use of systemic minocycline for over 20 years.

## Case presentation

A 66-year-old male presented to the clinic for a yearly follow-up skin examination. His dermatologic history included rosacea, disseminated superficial actinic porokeratosis (DSAP), and non-melanoma skin cancer. He had been taking minocycline 100 milligrams daily over the past 22 years with no reported side effects. Prior topical treatments for his rosacea included metronidazole gel and clindamycin lotion.

**Figure 1:**
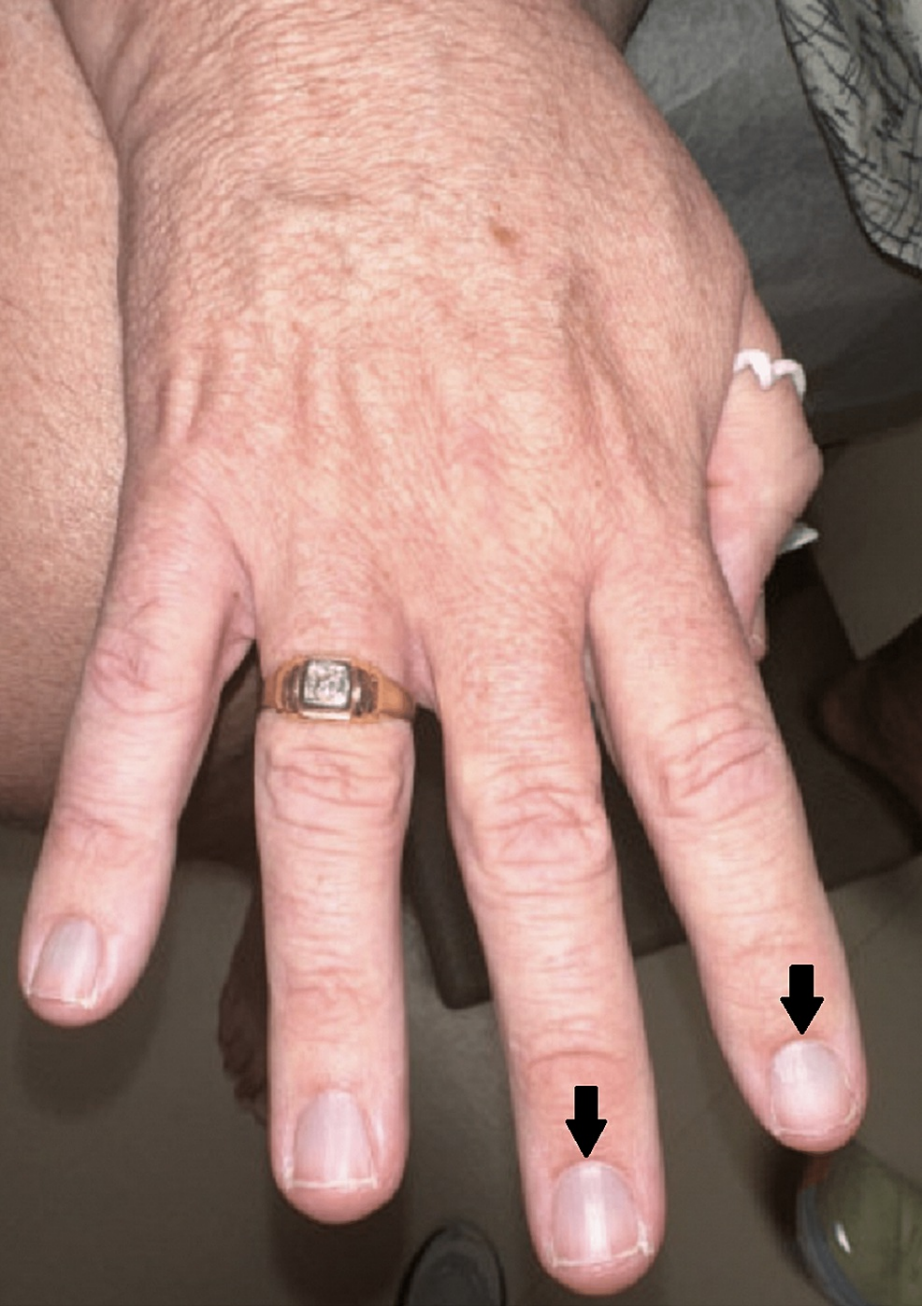
Minocycline-induced hyperpigmentation of nail beds. Greater blue-gray hyperpigmentation over the nail bed is appreciable in the right hand.

**Figure 2:**
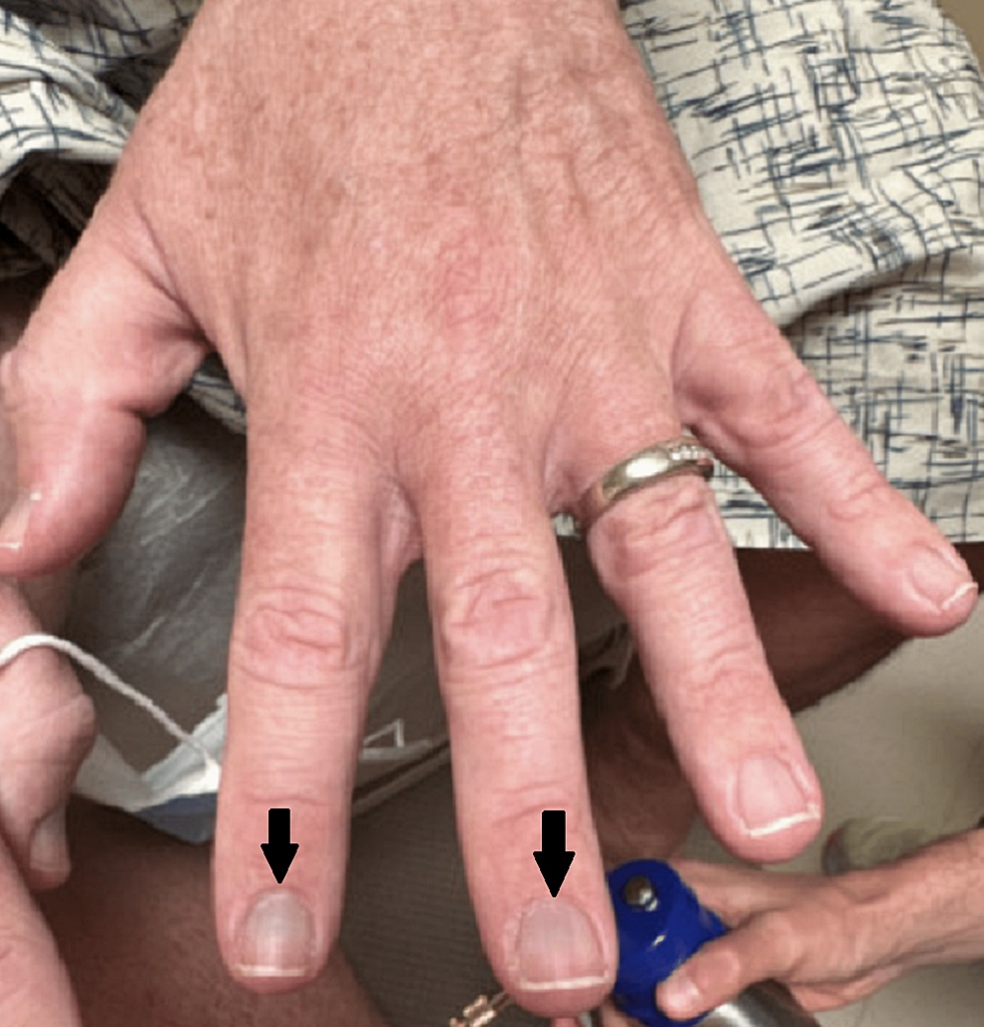
Minocycline-induced hyperpigmentation. Blue-gray hyperpigmentation of the fingernails is seen in the left hand.

On examination, the patient had erythema and telangiectasias of the dorsal nose, nasal sidewalls, and cheeks with a few scattered pustules. Mild rhinophyma of the nose was noted. He denied any ocular symptoms. A review of systems for additional constitutional, cardiac, pulmonary, and gastrointestinal symptoms was unremarkable. Upon examination of the fingernails, blue-gray hyperpigmentation of the nail beds bilaterally was observed (Figures 1, 2). Examination of the sclera, oral cavity, and remaining cutaneous surfaces was unremarkable for hyperpigmentation elsewhere.

The patient was informed that the blue hyperpigmentation of his nails was likely an adverse effect of his chronic minocycline use. His primary care physician had believed his nails to be discolored due to cardiac disease, but the patient reported no cardiac issues to his knowledge. When a substitute for minocycline was recommended, the patient was hesitant to do so. The patient had tried several other topical and systemic medications; however, he was adamant that minocycline gave him the greatest relief from his rosacea. The patient insisted upon the continuation of minocycline. The patient was counseled on the adverse effects of minocycline use and scheduled for follow-up.

## Discussion

Minocycline-induced hyperpigmentation is categorized into five types: type I with blue-black discoloration in areas of prior inflammation, such as scars secondary to acne; type II with blue-gray pigmentation of normal areas of skin, such as the anterior legs; type III with less common diffuse muddy brown hyperpigmentation worsened in sun-exposed areas; type IV with blue-gray macules in areas of scarring on the back; type V with hyperpigmentation and subcutaneous involvement, which is thought to represent a progression of types I and II into the subcutis [6]. The phenomenon of "blue nails" secondary to the use of minocycline is considered a type II hyperpigmentation. The incidence of types II and III hyperpigmentation increases with the cumulative dose of minocycline [6].

Many cases of blue nail discoloration secondary to minocycline use are associated with hyperpigmentation elsewhere. Other commonly affected areas include skin, teeth, mucous membranes, and sclera [2-5]. This is a unique case of blue-gray hyperpigmentation isolated to the nail beds with no other appreciable areas involved on the exam. This case of type II hyperpigmentation merits expansion of the variable presentations of minocycline-induced hyperpigmentation.

The treatment of type II minocycline-induced hyperpigmentation includes discontinuing the medication; areas involved may take months to years to fade to original pigmentation [7]. Further treatments include Q-switched and picosecond alexandrite lasers for persistent or cosmetically disturbing discolored areas [8,9]. Early recognition and evaluation for conditions with similar presentations, such as cyanosis, may be of benefit to the patient.

## Conclusions

Type II minocycline-induced hyperpigmentation is a benign side effect, the treatment for which includes discontinuing the medication. Here we present a unique case of blue-gray hyperpigmentation limited to the nail beds, without hyperpigmentation appreciable elsewhere. Early recognition of minocycline-induced hyperpigmentation and evaluation for conditions with similar presentations, such as cyanosis, may be of benefit to the patient. Patients should be counseled about this side effect of the medication and monitored.

## References

[1] Martins AM, Marto JM, Johnson JL, Graber EM (2021). A review of systemic minocycline side effects and topical minocycline as a safer alternative for treating acne and rosacea. Antibiotic.

[2] Tavares J, Leung WW (2011). Discoloration of nail beds and skin from minocycline. CMAJ.

[3] Ricardo JW, Shah K, Minkis K, Lipner SR (2022). Blue skin, nail, and scleral pigmentation associated with minocycline. Case Rep Dermatol.

[4] La Placa M, Infusino SD, Balestri R, Vincenzi C (2017). Minocycline-induced blue-gray discoloration. Skin Appendage Disord.

[5] Law S (2021). Minocycline-induced blue sclera and skin hyperpigmentation. BMJ Case Rep.

[6] Wang RF, Ko D, Friedman BJ, Lim HW, Mohammad TF (2023). Disorders of hyperpigmentation. Part I. Pathogenesis and clinical features of common pigmentary disorders. J Am Acad Dermatol.

[7] Kobayashi T, Hayakawa K (2017). Minocycline-induced skin pigmentation. Infection.

[8] Nisar MS, Iyer K, Brodell RT, Lloyd JR, Shin TM, Ahmad A (2013). Minocycline-induced hyperpigmentation: comparison of 3 Q-switched lasers to reverse its effects. Clin Cosmet Investig Dermatol.

[9] Barrett T, de Zwaan S (2018). Picosecond alexandrite laser is superior to Q-switched Nd:YAG laser in treatment of minocycline-induced hyperpigmentation: a case study and review of the literature. J Cosmet Laser Ther.

